# Transition probabilities of HER2-positive and HER2-negative breast cancer patients treated with Trastuzumab obtained from a clinical cancer registry dataset

**DOI:** 10.1016/j.dib.2016.03.039

**Published:** 2016-03-12

**Authors:** Monika Pobiruchin, Sylvia Bochum, Uwe M. Martens, Meinhard Kieser, Wendelin Schramm

**Affiliations:** a GECKO Institute for Medicine, Informatics and Economics, Heilbronn University, Max-Planck-Str. 39, 74081 Heilbronn, Germany; b Cancer Center Heilbronn-Franken, SLK-Hospitals, Am Gesundbrunnen 20-26, 74078 Heilbronn, Germany; c Institute of Medical Biometry and Informatics, University of Heidelberg, Im Neuenheimer Feld 305, 69120 Heidelberg, Germany

## Abstract

Records of female breast cancer patients were selected from a clinical cancer registry and separated into three cohorts according to HER2-status (human epidermal growth factor receptor 2) and treatment with or without Trastuzumab (a humanized monoclonal antibody). Propensity score matching was used to balance the cohorts. Afterwards, documented information about disease events (recurrence of cancer, metastases, remission of local/regional recurrences, remission of metastases and death) found in the dataset was leveraged to calculate the annual transition probabilities for every cohort.

**Specifications table**

**Table:** 

Subject area	*Medicine*
More specific subject area	*Oncology*, *Health Services Research*
Type of data	*Table*
How data was acquired	*Retrospective analysis*
*Database export of clinical cancer registry*
Data format	*Filtered*
Experimental factors	*Selection of matching cases from the cancer registry according to the study protocol by*[1]
Experimental features	*Data includes occurrences of disease events and calculated transition probabilities.*
Data source location	*Cancer Center at SLK*-*Hospitals*, *Heilbronn*, *Germany*
Data accessibility	*Data is with this article*

**Value of the data**

• Numbers of events (recurrences, metastases and death) are based on a patient cohort collected in a routine care environment, i.e., real world data.
• Comparison of raw numbers could serve as benchmarks for other cancer registries, hospitals, etc.
• Transition probabilities are estimated based on real world data only and could be used in other health economic Markov models.
• Transition probabilities could be utilized for validation procedures of other health economic models.

## 1 Data

We observed the disease progress for HER2-positive and HER2-negative patients in a routine care setting, i.e., real world setting.

Data is presented twofold:

1) Number of patients which shift from a defined health state (*Disease free*, *Recurrence*, *Metastasis*, *Remission recurrence*, *Remission metastasis*, *Death*) to another.
2) Transition probabilities for every year over a time horizon of *H*=8 years.

Numbers and transition probabilities are reported for every cohort:

C-1: HER2-positive patients/not treated with Trastuzumab
C-2: HER2-positive patients/treated with Trastuzumab
C-3: HER2-negative patients/not treated with Trastuzumab.

## 2 Experimental design, materials and methods

### 2.1 Patients

Our patient cohort comprised *n*=3230 cases of female breast cancer diagnosed from 01-01-2004 till 31-12-2012 and documented at the clinical cancer registry of the Cancer Center Heilbronn-Franken (CC). Patients were included in the cohort according to the HERA (Herceptin Adjuvant Trial)-study protocol׳s inclusion/exclusion criteria [1] as far as the criteria were applicable to the local documentation setting. This yielded 892 matching cases.

Afterwards patients were separated according to HER2-status and treatment with Trastuzumab.

This cohort was separated into four subcohorts C-1 (positive HER2-status and no Trastuzumab treatment), C-2 (positive HER2-status and Trastuzumab treatment), C-3 (negative HER2-status and no Trastuzumab treatment) and C-4 (negative HER2-status and Trastuzumab treatment). However, cohort C-4 needed to be excluded from further analyses, since from a clinical point of view it is not appropriate to treat HER2-negative patients with Trastuzumab. We assume that there are either misclassifications or documentation errors in these three records.

### 2.2 Propensity score matching

A first patient characteristics analysis of the cohorts C-1 to C-3 revealed that there were differences with respect to the distribution of age, tumor sizes, hormone receptor status, etc. Therefore, we balanced cohorts C-1 to C-3 with the propensity score matching method [2]. For this step, we used the MatchIt-package for the statistical software R which implements the nearest neighbor method [3]. Cohort C-2 served as reference population for the matching process. After this step, every cohort comprised 138 cases.

### 2.3 Database extraction

Several health states (*Disease free*, *Recurrence*, *Metastasis*, *Remission recurrence*, *Remission metastasis*, *Death*) were defined beforehand according to a reference study by Blank et al. [4]. These definitions were used to automatically generate SQL (Structured Query Language) scripts which extracted the patients׳ events. Thus raw numbers for the occurrence of several events (or states), e.g., getting a metastasis or death, could be determined. For a detailed description on how disease state information were mapped against the local tumor documentation system, the generation of SQL scripts and the processing of the results please refer to the research article for this data article [5].

### 2.4 Estimation of transition probabilities

Based on the extracted health state information and the patients׳ transitions between these states, maximum likelihood estimation of the transition matrix for the probability of any shift was performed [6] and compared to probabilities used in the model generated by [4]. Thus, the transition probabilities presented in the Supplementary material (Table 1) of this article were calculated.

## Footnotes

Appendix A Supplementary data associated with this article can be found in the online version at 10.1016/j.dib.2016.03.039.
