# Supplementary material for: Transition probabilities of HER2-positive and HER2-negative breast cancer patients treated with Trastuzumab obtained from a clinical cancer registry dataset
Source: Data Brief. 2016 Mar 12;7:654–7. doi: 10.1016/j.dib.2016.03.039 (PMC4802671; doi:10.1016/j.dib.2016.03.039)
Supplement: Supplementary file 1 — Supplementary material [file mmc1.docx]

***Conflict of Interests:*** We wish to confirm that there are no known conflicts of interest associated with this publication and there has been no significant financial support for this work that could have influence its outcome.
