# Supplementary material for: Transition probabilities of HER2-positive and HER2-negative breast cancer patients treated with Trastuzumab obtained from a clinical cancer registry dataset
Source: Data Brief. 2016 Mar 12;7:654–7. doi: 10.1016/j.dib.2016.03.039 (PMC4802671; doi:10.1016/j.dib.2016.03.039)
Supplement: Supplementary file 2 — Supplementary material [file mmc2.zip › dib_article_supplement.docx]

Appendix: Transition Matrices

| **Cycle c_t_** | **1** | **2** | | **3** | | **4** | | **5** | | **6** | | **7** | | **8** |
| --- | --- | --- | --- | --- | --- | --- | --- | --- | --- | --- | --- | --- | --- | --- |
| *Disease free* 🡪 *Disease free* |  |  | |  | |  | |  | |  | |  | |  |
| C-1:HER2+/No Trastuzumab | 92.6  126 | 92.9  117 | | 94.9  111 | | 99.1  110 | | 100  110 | | 99.1  109 | | 100  109 | | 96.3  105 |
|  | (87.9) | | | | | | | | | | | | | |
| C-2: HER2+/Trastuzumab | 96.1  124 | 97.6  121 | | 97.5  118 | | 99.2  117 | | 98.3  115 | | 100  115 | | 100  115 | | 99.1  114 |
|  | (93.4) | | | | | | | | | | | | | |
| C-3:HER2-/No Trastuzumab | 93.4  127 | 97.6  124 | | 94.4  117 | | 100  117 | | 98.3  115 | | 97.4  112 | | 98.2  110 | | 99.1  109 |
|  | (97.5) | | | | | | | | | | | | | |
| *Disease free* 🡪 *Recurrence* |  |  | |  | |  | |  | |  | |  | |  |
| C-1:HER2+/No Trastuzumab | 2.2  3 | 1.6  2 | | 0.9  1 | | 0.9  1 | | 0.0  0 | | 0.9  1 | | 0.0  0 | | 0.9  1 |
|  | (3.0) | | | | | | | | | | | | | |
| C-2: HER2+/Trastuzumab | 2.3  3 | 0.0  0 | | 2.5  3 | | 0.0  0 | | 0.9  1 | | 0.0  0 | | 0.0  0 | | 0.9  1 |
|  | (1.6) | | | | | | | | | | | | | |
| C-3:HER2-/No Trastuzumab | 1.5  2 | 0.8  1 | | 1.6  2 | | 0.0  0 | | 0.0  0 | | 1.7  2 | | 1.8  2 | | 0.0  0 |
|  | (0.8) | | | | | | | | | | | | | |
| *Disease free* 🡪 *Death* |  | | | | | | | | | | | | | |
| C-1:HER2+/No Trastuzumab | 0.07  1 | | 1.6  2 | | 0.9  1 | | 0  0 | | 0  0 | 0  0 | 0  0 | | 0  0 | |
|  | (na) | | | | | | | | | | | | | |
| C-2: HER2+/Trastuzumab | 0.08  1 | | 0.08  1 | | 0  0 | | 0  0 | | 0.09  1 | 0  0 | 0  0 | | 0  0 | |
|  | (na) | | | | | | | | | | | | | |
| C-3:HER2-/No Trastuzumab | 1.5  2 | | 0  0 | | 2.4  3 | | 0  0 | | 0.9  1 | 0  0 | 0  0 | | 0.9  1 | |
|  | (na) | | | | | | | | | | | | | |

| **Cycle c_t_** | **1** | **2** | | **3** | | **4** | | **5** | | **6** | | **7** | | **8** |
| --- | --- | --- | --- | --- | --- | --- | --- | --- | --- | --- | --- | --- | --- | --- |
| *Disease free* 🡪 *Metastasis* |  |  | |  | |  | |  | |  | |  | |  |
| C-1:HER2+/No Trastuzumab | 4.4  6 | 4.0  5 | | 3.4  4 | | 0.0  0 | | 0.0  0 | | 0.0  0 | | 0.0  0 | | 2.8  3 |
|  | (9.1) | | | | | | | | | (8.2) | | (7.4) | | (6.6) |
| C-2: HER2+/Trastuzumab | 0.8  1 | 1.6  2 | | 0.0  0 | | 0.8  1 | | 0.0  0 | | 0.0  0 | | 0.0  0 | | 0.0  0 |
|  | (5.0) | | | | | | | | | (4.5) | | (4.1) | | (3.6) |
| C-3:HER2-/No Trastuzumab | 3.7  5 | 1.6  2 | | 1.6  2 | | 0.0  0 | | 0.9  1 | | 0.9  1 | | 0.0  0 | | 0.0  0 |
|  | (1.7) | | | | | | | | | (1.5) | | (1.4) | | (1.2) |
| *Recurrence 🡪 Recurrence* | | | | | | | | | | | | | | |
| C-1:HER2+/No Trastuzumab | 0  0 | | 33.3  1 | | 0  0 | | 50.0  1 | | 100  3 | 100  3 | | 75.0  3 | | 100  3 |
|  | (na) | | | | | | | | | | | | | |
| C-2: HER2+/Trastuzumab | 0  0 | | 0  0 | | 0  0 | | 0  0 | | 0  0 | 100  1 | | 100  1 | | 100  1 |
|  | (na) | | | | | | | | | | | | | |
| C-3:HER2-/No Trastuzumab | 0  0 | | 50.0  1 | | 50.0  1 | | 0  0 | | 0  0 | 0  0 | | 0  0 | | 50.0  1 |
|  | (na) | | | | | | | | | | | | | |
| *Recurrence 🡪 Remission recurrence* | | | | | | | | | | | | | | |
| C-1:HER2+/No Trastuzumab | 100  1 | | 33.3  1 | | 20.0  1 | | 0  0 | | 0  0 | 0  0 | 25.0  1 | | 0  0 | |
|  | (na) | | | | | | | | | | | | | |
| C-2: HER2+/Trastuzumab | 0  0 | | 33.3  1 | | 0  0 | | 25.0  1 | | 0  0 | 0  0 | 0  0 | | 0  0 | |
|  | (na) | | | | | | | | | | | | | |
| C-3:HER2-/No Trastuzumab | 0  0 | | 100  1 | | 50.0  1 | | 33.3  1 | | 0  0 | 0  0 | 0  0 | | 0  0 | |
|  | (na) | | | | | | | | | | | | | |

| **Cycle c_t_** | **1** | **2** | | **3** | | **4** | | **5** | | **6** | | **7** | | **8** |
| --- | --- | --- | --- | --- | --- | --- | --- | --- | --- | --- | --- | --- | --- | --- |
| *Recurrence 🡪 Death* | | | | | | | | | | | | | | |
| C-1:HER2+/No Trastuzumab | 0  0 | | 0  0 | | 60.0  3 | | 0  0 | | 0  0 | 0  0 | | 0  0 | | 0  0 |
|  | (na) | | | | | | | | | | | | | |
| C-2: HER2+/Trastuzumab | 0  0 | | 0  0 | | 0  0 | | 25.0  1 | | 0  0 | 0  0 | | 0  0 | | 0  0 |
|  | (na) | | | | | | | | | | | | | |
| C-3:HER2-/No Trastuzumab | 0  0 | | 0  0 | | 0  0 | | 0  0 | | 0  0 | 0  0 | | 0  0 | | 0  0 |
|  | (na) | | | | | | | | | | | | | |
| *Remission recurrence 🡪 Remission recurrence* | | | | | | | | | | | | | | |
| C-1:HER2+/No Trastuzumab | 0  0 | | 100  1 | | 100  2 | | 100  3 | | 66.7  2 | 100  2 | 100  2 | | 66.7  2 | |
|  | (na) | | | | | | | | | | | | | |
| C-2: HER2+/Trastuzumab | 0  0 | | 0  0 | | 100  1 | | 100  1 | | 100  2 | 100  2 | 100  2 | | 100  2 | |
|  | (na) | | | | | | | | | | | | | |
| C-3:HER2-/No Trastuzumab | 0  0 | | 0  0 | | 100  1 | | 50.0  1 | | 100  2 | 50.0  1 | 100  1 | | 100  1 | |
|  | (na) | | | | | | | | | | | | | |
| *Remission recurrence 🡪 Metastasis* | | | | | | | | | | | | | | |
| C-1:HER2+/No Trastuzumab | 0  0 | | 0  0 | | 0  0 | | 0  0 | | 33.3  1 | 0  0 | 0  0 | | 33.3  1 | |
|  | (na) | | | | | | | | | | | | | |
| C-2: HER2+/Trastuzumab | 0  0 | | 0  0 | | 0  0 | | 0  0 | | 0  0 | 0  0 | 0  0 | | 0  0 | |
|  | (na) | | | | | | | | | | | | | |
| C-3:HER2-/No Trastuzumab | 0  0 | | 0  0 | | 0  0 | | 50.0  1 | | 0  0 | 50.0  1 | 0  0 | | 0  0 | |
|  | (na) | | | | | | | | | | | | | |

| **Cycle c_t_** | **1** | **2** | | **3** | | **4** | | **5** | | **6** | | **7** | | **8** |
| --- | --- | --- | --- | --- | --- | --- | --- | --- | --- | --- | --- | --- | --- | --- |
| *Metastasis 🡪 Metastasis* | | | | | | | | | | | | | | |
| C-1:HER2+/No Trastuzumab | 0  0 | | 16.7  1 | | 42.9  3 | | 50.0  4 | | 80.0  4 | 80.0  4 | 75.0  3 | | 100  3 | |
|  | (67.0) | | | | | | | | | (60.3) | (54.3) | | (48.9) | |
| C-2: HER2+/Trastuzumab | 0  0 | | 0  0 | | 25.0  1 | | 100  1 | | 50.0  2 | 100  2 | 100  2 | | 100  2 | |
|  | (78.0) | | | | | | | | | (70.2) | (63.2) | | (56.9) | |
| C-3:HER2-/No Trastuzumab | 0  0 | | 40.0  2 | | 50.0  2 | | 50.0  2 | | 60.0  3 | 100  4 | 66.7  4 | | 100  6 | |
|  | (78.8) | | | | | | | | | (70.9) | (63.8) | | (57.4) | |
| *Metastasis 🡪 Remission metastasis* | | | | | | | | | | | | | | |
| C-1:HER2+/No Trastuzumab | 0  0 | | 0  0 | | 0  0 | | 0  0 | | 0  0 | 0  0 | 0  0 | | 0  0 | |
|  | (na) | | | | | | | | | | | | | |
| C-2: HER2+/Trastuzumab | 0  0 | | 0  0 | | 0  0 | | 0  0 | | 0  0 | 0  0 | 0  0 | | 0  0 | |
|  | (na) | | | | | | | | | | | | | |
| C-3:HER2-/No Trastuzumab | 0  0 | | 0  0 | | 0  0 | | 0  0 | | 0  0 | 0  0 | 16.7  1 | | 0  0 | |
|  | (na) | | | | | | | | | | | | | |
| *Metastasis 🡪 Death* | | | | | | | | | | | | | | |
| C-1:HER2+/No Trastuzumab | 0  0 | | 50.0  3 | | 42.9  3 | | 37.5  3 | | 20.0  1 | 20.0  1 | 25.0  1 | | 0  0 | |
|  | (33.0) | | | | | | | | | (39.7) | (45.7) | | (51.1) | |
| C-2: HER2+/Trastuzumab | 0  0 | | 100  1 | | 50.0  2 | | 0  0 | | 50.0  2 | 0  0 | 0  0 | | 0  0 | |
|  | (22.0) | | | | | | | | | (29.8) | (36.8) | | (43.1) | |
| C-3:HER2-/No Trastuzumab | 0  0 | | 60.0  3 | | 50.0  2 | | 50.0  2 | | 40.0  2 | 0  0 | 16.7  1 | | 0  0 | |
|  | (21.2) | | | | | | | | | (29.1) | (36.2) | | (42.6) | |

| **Cycle c_t_** | **1** | **2** | | **3** | | **4** | | **5** | | **6** | | **7** | | **8** |
| --- | --- | --- | --- | --- | --- | --- | --- | --- | --- | --- | --- | --- | --- | --- |
| *Remission metastasis 🡪 Remission metastasis* | | | | | | | | | | | | | | |
| C-1:HER2+/No Trastuzumab | 0  0 | | 0  0 | | 0  0 | | 0  0 | | 0  0 | 0  0 | 0  0 | | 0  0 | |
|  | (na) | | | | | | | | | | | | | |
| C-2: HER2+/Trastuzumab | 0  0 | | 0  0 | | 0  0 | | 0  0 | | 0  0 | 0  0 | 0  0 | | 0  0 | |
|  | (na) | | | | | | | | | | | | | |
| C-3:HER2-/No Trastuzumab | 0  0 | | 0  0 | | 0  0 | | 0  0 | | 0  0 | 0  0 | 0  0 | | 100  1 | |
|  | (na) | | | | | | | | | | | | | |
| *Death 🡪 Death* | | | | | | | | | | | | | | |
| C-1:HER2+/No Trastuzumab | 0  0 | | 100  1 | | 100  6 | | 100  13 | | 100  16 | 100  17 | 100  18 | | 100  19 | |
|  | (na) | | | | | | | | | | | | | |
| C-2: HER2+/Trastuzumab | 0  0 | | 100  1 | | 100  3 | | 100  5 | | 100  6 | 100  9 | 100  9 | | 100  9 | |
|  | (na) | | | | | | | | | | | | | |
| C-3:HER2-/No Trastuzumab | 0  0 | | 100  2 | | 100  5 | | 100  10 | | 100  12 | 100  15 | 100  15 | | 100  15 | |
|  | (na) | | | | | | | | | | | | | |

Table 1: Annual transition probabilities generated for the Cancer Center dataset and the number of transitions observed. Values given by Blank et al. [4] are shown in (). Na = Not available, i.e., probability not given in the respective model. For instance, probabilities for recurrences were given for local and regional recurrences in [4].
